# Cardiac Optogenetics and Optical Mapping – Overcoming Spectral Congestion in All-Optical Cardiac Electrophysiology

**DOI:** 10.3389/fphys.2019.00182

**Published:** 2019-03-07

**Authors:** Christopher O’Shea, Andrew P. Holmes, James Winter, Joao Correia, Xianhong Ou, Ruirui Dong, Shicheng He, Paulus Kirchhof, Larissa Fabritz, Kashif Rajpoot, Davor Pavlovic

**Affiliations:** ^1^Institute of Cardiovascular Sciences, University of Birmingham, Birmingham, United Kingdom; ^2^School of Computer Science, University of Birmingham, Birmingham, United Kingdom; ^3^EPSRC Centre for Doctoral Training in Physical Sciences for Health, School of Chemistry, University of Birmingham, Birmingham, United Kingdom; ^4^Institute of Clinical Sciences, University of Birmingham, Birmingham, United Kingdom; ^5^Institute of Microbiology and Infection, School of Biosciences, University of Birmingham, Birmingham, United Kingdom; ^6^Key Laboratory of Medical Electrophysiology of Ministry of Education, Collaborative Innovation Center for Prevention and Treatment of Cardiovascular Disease/Institute of Cardiovascular Research, Southwest Medical University, Luzhou, China; ^7^Department of Cardiology, UHB NHS Trust, Birmingham, United Kingdom

**Keywords:** optogenetic, optical mapping, fluorescence, cardiac, action potential, calcium, conduction (action potential), arrhythmias

## Abstract

Optogenetic control of the heart is an emergent technology that offers unparalleled spatio-temporal control of cardiac dynamics via light-sensitive ion pumps and channels (opsins). This fast-evolving technique holds broad scope in both clinical and basic research setting. Combination of optogenetics with optical mapping of voltage or calcium fluorescent probes facilitates ‘all-optical’ electrophysiology, allowing precise optogenetic actuation of cardiac tissue with high spatio-temporal resolution imaging of action potential and calcium transient morphology and conduction patterns. In this review, we provide a synopsis of optogenetics and discuss in detail its use and compatibility with optical interrogation of cardiac electrophysiology. We briefly discuss the benefits of all-optical cardiac control and electrophysiological interrogation compared to traditional techniques, and describe mechanisms, unique features and limitations of optically induced cardiac control. In particular, we focus on state-of-the-art setup design, challenges in light delivery and filtering, and compatibility of opsins with fluorescent reporters used in optical mapping. The interaction of cardiac tissue with light, and physical and computational approaches to overcome the ‘spectral congestion’ that arises from the combination of optogenetics and optical mapping are discussed. Finally, we summarize recent preclinical work applications of combined cardiac optogenetics and optical mapping approach.

## Introduction

Over the past 30 years high resolution camera technologies and development of several potentiometric and intracellular calcium sensors has led to optical mapping becoming a valuable tool in cardiac research (Salama et al., 1987; Boukens and Efimov, 2014). Electrical conduction, action potential and calcium transient morphology can be directly measured, quantified, and tracked across multicellular cardiac preparations in high spatio-temporal resolution, unparalleled by traditional electrode techniques (Herron et al., 2012; Yu et al., 2014). Optical mapping has hence played a pivotal role in cardiac research, providing several insights into physiology and pathophysiology of the heart (Jalife, 2003; Myles et al., 2012; Syeda et al., 2016; Winter et al., 2018).

Conversely, optogenetics shifts light to an actuator role to control and tune EP behavior through genetically introducing photosensitive ion channels and pumps (opsins), able to depolarize and hyperpolarize excitable cells. This emergent technology, with its foundations in neuroscience, is now increasingly exploited by heart researchers. Optogenetic pacing of cardiac preparations has now been reported in several experimental models (Park et al., 2014; Vogt et al., 2015; Zhu et al., 2016; Nyns et al., 2017). Beyond rhythm control, optogenetics has been used to terminate arrhythmias in both *ex vivo* and *in vivo* rodent hearts (Bruegmann et al., 2016, 2018; Nyns et al., 2017), suppress and manipulate rotors in cardiomyocyte monolayers (Feola et al., 2017; Watanabe et al., 2017) and elucidate the function of both cardiomyocyte (Wang et al., 2017) and non-cardiomyocyte (Hulsmans et al., 2017) cellular subpopulations in the heart. Hence, optogenetics presents a pivotal emergent technology for basic research, while some of its unique features make it a potentially transformative clinical tool for both pacing and arrhythmia termination.

**FIGURE 1:**
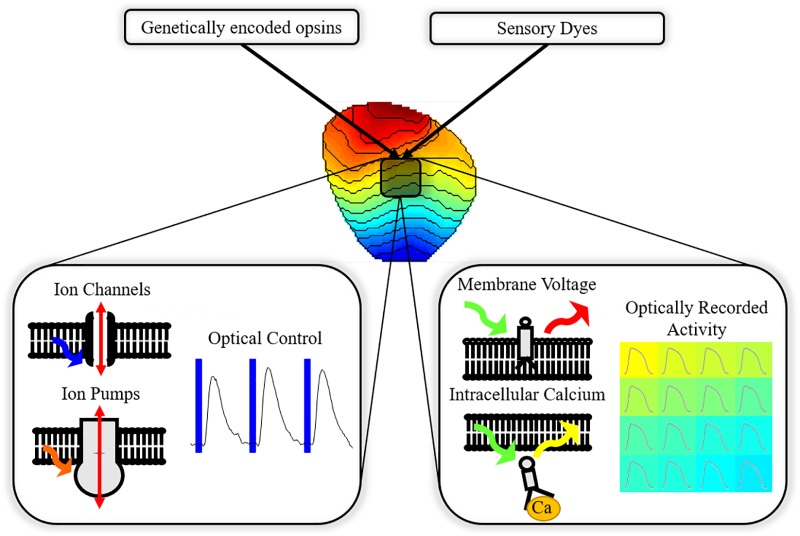
All-optical electrophysiology. Cardiac preparations treated with opsins and sensors allow simultaneous optically driven control of the heart (pacing or modulation of the action potential) and optical recording of action potential or calcium handling across the myocardium.

Combination of optogenetics with optical imaging of cellular monolayers and whole hearts allows ‘all-optical’ cardiac EP investigation (Figure 1); an approach that has seen growing application in cardiac research (Entcheva, 2013; Nussinovitch and Gepstein, 2015b; Entcheva and Bub, 2016). Owing to contactless operation and high spatio-temporal resolution, all-optical systems are uniquely capable of high throughput control and study of complex phenomena that can arise in excitable media. High spatio-temporal understanding of cardiac behavior in response to optical actuation, aside from providing an invaluable research tool, is also vital if optogenetics is to transition to clinical utility.

The requirement and employment of all-optical approach has been expansively presented in recent reviews (Entcheva and Bub, 2016; Crocini et al., 2017). In the present article, we discuss the basic tools of optogenetics (opsins), and optical mapping (voltage and calcium sensors). We focus on their dual use in all-optical setups, including their mechanisms of action, delivery to cardiac tissue, spectral compatibility and highlight technical considerations and advances that have made all-optical systems practical. Additionally, benefits of all-optical cardiac control and EP interrogation when compared to traditional contact techniques are discussed, and we highlight recent applications of combined cardiac optogenetics and optical mapping approaches.

## Tools of Optogenetics – Opsins

**FIGURE 2:**
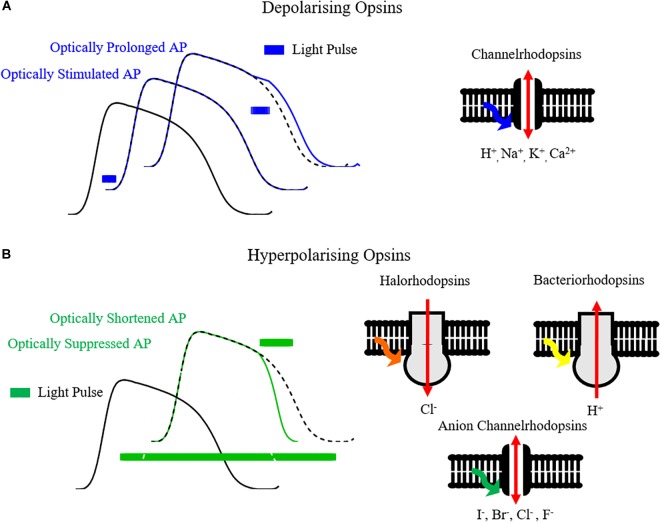
Optogenetic control of the cardiac action potential. **(A)** Opsins such as channelrhodopsins, by conducting cations such as H^+^_,_ Na^+^_,_ K^+^_,_ Ca^2+^ on light activation, can depolarize the cell membrane and hence initiate or prolong the cardiac action potential. **(B)** Hyperpolarizing opsins such as Halorhodopsins (Cl^-^ pumps), Bacteriorhodopsins (H^+^ pumps) and Anion Channelrhodopsins (I^-^, Br^-^, Cl^-^_,_ F^-^ channels) can shorten or completely suppress the action potential.

The development of optogenetics as a valuable research tool stems from the discovery and cloning of microbial opsins that behave as light-gated ionic channels in the early to mid-2000s – Channelrhodopsins (Nagel et al., 2003, 2005; Boyden et al., 2005). In particular Channelrhodopsin2 (ChR2), and variants thereof, is by far the most utilized opsin in optogenetics. On excitation by blue light (∼470 nm) of threshold irradiance, ChR2 opens as its covalently bound photosensitive chromophore, all-trans retinal, isomerizes (Nagel et al., 2003). The opening of the channel allows ions, including Na^+^, to cross the cellular membrane, as occurs in phase 0 of the action potential, initiating depolarization. Hence, cells expressing ChR2 can be effectively stimulated with blue light, initiating and/or prolonging the action potential (Figure 2A). As well as being light sensitive, ChR2 also acts in a voltage dependent manner, with decreasing conductance at more positive membrane potentials and a reversal potential near 0mV (Lin, 2010; Park et al., 2014).

Enhancement of the native properties of ChR2 is vital for the future use of optogenetics in cardiac tissue, due to relatively small currents generated by wild type ChR2 (steady state current ∼0.25 nA) and large absorption and scattering of visible light by biological chromophores. This limits optical penetration depth, preventing transmural opsin activation from deep tissue. Deep activation would be advantageous for synchronous cardiac activation and has been demonstrated as an important factor in successful re-entry termination (Karathanos et al., 2016; Watanabe et al., 2017). One possible method to realize deep tissue activation is red-shifting of the excitation window toward the ‘biological window’ of 650–1350 nm, where the extinction coefficient of biological tissue is greatly reduced (Smith et al., 2009). Light in this wavelength range can penetrate a few centimeters into the cardiac tissue and thus more light can reach and activate deep tissue opsins. Furthermore, decreasing the threshold irradiance required for photocurrent activation and/or increasing channel conductance or opening times, can aid deep tissue activation and reduce energy requirements in optogenetic applications.

Hence, since its isolation and cloning, ChR2 has undergone several alterations including variants with enhanced conductance [ChR2-H134R (Nagel et al., 2005), ChR2-T159C (Berndt et al., 2011), ChR2-XXL (Dawydow et al., 2014)], Ca^2+^ permeability [CatCh (Kleinlogel et al., 2011)] and red shifted spectral properties [ReaChR (Lin et al., 2013)]. Additionally, channelrhodopsins separate from ChR2 have been cloned from distinct algae species and subsequently optimized [CheRiff (Hochbaum et al., 2014)], while chimeras of ChR1 and ChR2 have also demonstrated enhanced photocurrents [ChIEF (Lin et al., 2009)]. These developments have led to a plethora of available depolarizing opsins with diverse spectral and kinetic properties (Schneider et al., 2015). Equally, it has been shown how exogenously supplemented all-trans retinal can significantly increase light sensitivity of ChR2, although concentration dependent cytotoxic effects were also observed (Yu et al., 2015).

An important factor in enhancing the photocurrent of native Channelrhodopsins is the effects mutagenesis can have on the photocycle kinetics. By prolonging channel opening times after the cessation of light activation, light sensitivity can be improved by a short pulse leading to a long-lasting photocurrent, e.g., ChR2-D156A which exhibits a dark off time of > 150 s (Bamann et al., 2010). However, significant slowing of the channel kinetics is detrimental to dynamic initiation and control of membrane perturbation. Therefore, the channelrhodopsins that have found most use in all optical setups (ChR2-H134R, CatCh, ReaChR, and CheRiff) are those whose enhanced photocurrents have been achieved with only moderate prolongation compared to wild type ChR2, which exhibits on and off time constants of ∼0.2 and ∼10 ms respectively (Nagel et al., 2003).

Aside from depolarizing Channelrhodopsins, hyperpolarizing opsin pumps include halorhodopsin Cl^-^ pumps (e.g., eNpHR3.0) and bacteriorhodopsin proton pumps (e.g., Arch and ArchT) (Kandori, 2015). Hyperpolarizing opsins allow selective suppression and shortening of action potentials (Figure 2B), and have been utilized for applications such as suppressing *in vivo* cardiac motion for high resolution imaging in zebrafish (Mickoleit et al., 2014). Thanks to distinct absorption characteristics in comparison with ChR2, a cardiac preparation expressing both depolarizing and hyperpolarizing opsins can be selectively stimulated and silenced simply by tuning illumination wavelength, thereby allowing comprehensive control of the action potential. While opsins can be expressed independently, gene-fusion enables formation of protein complexes such as ChR2-ArchT, allowing co-localization and bi-directional control of membrane voltage with one protein complex (Nussinovitch et al., 2014; Streit and Kleinlogel, 2018).

Hyperpolarizing pumps suffer from restricted photocurrent as one ion is transported per absorbed photon (Berndt et al., 2011). Anion channelrhodopsins (ACRs), with the potential for enhanced conductance driven instead by electrochemical gradients, were first realized by mutation of channelrhodopsins to infer Cl^-^ conductance, although with some remaining cationic conductance and slowing of the channel kinetics (Berndt et al., 2014; Wietek et al., 2014). Subsequently, naturally occurring ACRs with reduced cationic conductance have been discovered and cloned (Govorunova et al., 2015). Preliminary studies indicate that reduced light intensities are required to optically induce hyper- and repolarizing currents in cardiomyocytes using these novel ACRs, compared to pumps (Govorunova et al., 2016). Greater structural and functional understanding of both natural (Kim et al., 2018) and designed (Kato et al., 2018) ACRs promises further optimization of inhibitory channels.

Prolonged use and activation of inhibitory pumps or channels based on conductance of H^+^ or Cl^-^ can detrimentally alter intracellular ionic concentrations (Alfonsa et al., 2015; Bernal Sierra et al., 2018). One promising avenue to potentially circumvent this limitation is the development of tools which more closely mimic the resting and repolarization mechanisms of excitable cells via light activated K^+^ conductance. Several approaches have now been tested, including recently published PAC-K constructs which combine a cAMP-gated K^+^ channel with photo-activated nucleotidyl cyclase, allowing optical silencing of cardiomyocyte (and neuron) activity (Bernal Sierra et al., 2018). These constructs, however, do not currently allow for fast responsive control of membrane potential, eliciting hyperpolarization lasting minimally for 100 ms, preventing dynamic control during the action potential.

### Delivery of Opsins in Cardiac Tissue

Optogenetic control requires reliable, rapidly responsive, and reversible generation of depolarizing and hyperpolarizing currents by the expression of the light gated ionic transport proteins. The first barrier to achieving cardiac optogenetic perturbation therefore is effective delivery and expression of desired opsins in cardiac preparations. Pioneering cardiac optogenetic studies in 2010 transgenically expressed ChR2 in zebrafish (Arrenberg et al., 2010) and mouse (Bruegmann et al., 2010). ChR2 expressing mouse lines remain prevalent (Quiñonez Uribe et al., 2018) and interventions such as Cre recombinase allow powerful research strategies (Wang et al., 2017).

However, requirement for transgenic expression of opsins is costly, time-consuming and limits clinical application of optogenetics (Knollmann, 2010). Therefore, other techniques have been explored exploiting tandem-cell-unit (Jia et al., 2011) and viral (Abilez et al., 2011; Vogt et al., 2015) delivery of ChR2 and other opsins. Tandem-cell-unit delivery centers on the concept where previously non-excitable cells, transfected to express ChR2, are grafted into cardiomyocyte preparations. The cells couple to the cardiomyocytes via gap junctional proteins and act as ‘sparks,’ initiating depolarization of coupled cardiomyocytes on light stimulation (Jia et al., 2011).

In viral delivery, opsin genes are encoded in lentiviruses, adenoviruses, or adeno-associated viruses (AAV). Viral methods allow for tissue or cell selectivity, depending on the promoter used, and can be directly injected to realize light excitability. Importantly for future clinical utility of optogenetics, there is increasing evidence that AAVs can be safely and efficaciously used in the heart (Bera and Sen, 2017) and systemic viral delivery can be used to promote cardiac specific ChR2 expression *in vivo* (Ambrosi et al., 2019). In wild type mice for example, AAV injection has shown to result in stable and long lasting ventricular (Vogt et al., 2015; Bruegmann et al., 2016) and atrial (Bruegmann et al., 2018) expression of ChR2. However, transfection rates and consequently optical sensitivity remain variable between treated hearts, and there is some evidence of chamber discrepancies with atrial transfection rates lower than ventricular (Vogt et al., 2015; Bruegmann et al., 2016, 2018). Hence, extensive effort is ongoing to realize the most effective method to introduce optical excitability to cardiac tissue (Ambrosi et al., 2015).

Reports that high-level expression of lentiviral-delivered ChR2 in NRVMs is associated with cytotoxicity also need to be carefully considered if translational potential of this technology is to be fully realized. The mechanisms underpinning cytotoxicity are unclear, with Ca^2+^ overload and membrane damage being implicated (Li et al., 2017). Further work on mechanistic insight into cytotoxicity is required to ensure safe use of ChR2 and other opsins in cardiac optogenetics.

## Optical Voltage and Calcium Sensors

### Synthetic Sensors

A range of sensors are utilized in optical mapping to image transmembrane voltage, as well fluctuations in cytosolic and sarcoplasmic reticulum Ca^2+^ concentrations (Broyles et al., 2018). Synthetic sensors are small molecules that are most commonly introduced to *ex vivo* hearts via Langendorff perfusion, or via superfusion to both *in vitro* and *ex vivo* preparations.

The most popular voltage sensors are ‘fast’ synthetic styryl sensors, such as di-4-ANEPPS and rh-237, which embed within the plasma membrane, Figure 1. As the transmembrane voltage changes, for example during the cardiac action potential, these sensors exhibit fast responding (femto- to picosecond) spectrally shifted fluorescent output due to shifts in charge state and hence dipole energy levels (electrochromism) (Loew et al., 1978; Miller, 2016). Longpass filtering the emitted fluorescence beyond the emission spectra maximum therefore allows recording of optical signals exhibiting fluorescence intensity proportional to cellular membrane voltage changes, i.e., optical action potentials (OAP) (Herron et al., 2012).

On the other hand, intracellular Ca^2+^ sensors such as rhod-2AM are designed to internalize within the cell, Figure 1. They are commonly esterified to neutralize the charge, aiding intracellular uptake. The ester is then enzymatically cleaved once in the cell, leaving behind a Ca^2+^ chelator and a fluorophore. As Ca^2+^ is chelated, fluorescence output increases, and subsequently decreases upon dissociation, reporting the changing [Ca^2+^] in the intracellular space where the sensor localizes, either in the cytosol or sarcoplasmic reticulum (Jaimes et al., 2016).

### Genetically Encoded Sensors

Alternatively to small molecule synthetic sensors, voltage and calcium responsive protein sensors can be genetically encoded to achieve cell specific indicator expression (Liao et al., 2015; Quinn et al., 2016). These are collectively termed genetically encoded voltages/calcium indicators (GEVIs/GECIs), constructed by the fusion of voltage/Ca^2+^ sensitive and fluorescent proteins (Scanziani and Häusser, 2009). Genetic indicators can be used to independently measure voltage or Ca^2+^, but can also be fused to create dual voltage-calcium constructs such as CaViar (Hou et al., 2014) which combines QuasAr2 (GEVI) with GCaMP6f (GECI). Furthermore, co-expressed indicator and actuator pairs offer a unique ability to provide all-genetic, all-optical electrophysiological study (Chang et al., 2017). The “Optopatch” platform for example combines genetic indicators with CheRiff and has been utilized both in establishing all-optical mouse lines and as a high throughput cardiotoxicity screening platform for all-optical pacing with simultaneous voltage and intracellular calcium measurement (Hochbaum et al., 2014; Dempsey et al., 2016; Björk et al., 2017).

### Synthetic vs. Genetically Encoded Sensors

Genetic indicators rely on conformational changes to directly alter their fluorescence response or via eliciting processes such as Forster resonance energy transfer (FRET). Consequently, compared to the ‘fast’ small-molecule sensors, an important limiting factor of current genetically encoded indicators is their response times which are typically in the order of milliseconds (Kaestner et al., 2015). This can prove problematic when measuring the sub millisecond phenomena involved in cardiac depolarization, repolarization and calcium handling (Koopman et al., 2017). Indeed, OAPs recorded by genetic indicators exhibit a significantly altered morphology compared to simultaneously measured OAPs using syntenic sensors (Shinnawi et al., 2015). Coupled to this, the relatively straightforward application to *ex vivo* and *in vitro* preparations makes synthetic sensors more commonly utilized in optical mapping studies (Herron et al., 2012), and to date the pioneering all-optical setups (Table 1).

**Table 1:** Opsin and sensor combinations used in all-optical setups, with opsin excitation, sensor excitation, and sensor emission spectral characteristics from a specific study.

Opsin	Opsin Excitation λ (nm)	Sensor	Type	Excitation source	Sensor Excitation λ (nm)	Sensor Emission λ (nm)	Reference	Other studies using specified Opsin/Sensor combination
		**Voltage Sensors**		
ChR2 variants: ChR2-H134R, CatCh, CheRiff (Depolarizing)	470	di-4-ANBDQBS	Synthetic	LED	660 (655/40)	LP700	Klimas et al., 2016	Nussinovitch and Gepstein, 2015a; Yu et al., 2015; Feola et al., 2017; Majumder et al., 2018
		di-4-ANBDQPQ	Synthetic	LED	625 (640/40)	774/140	Crocini et al., 2016	Scardigli et al., 2018; Streit and Kleinlogel, 2018; Quiñonez Uribe et al., 2018
		PGH1	Synthetic	LED	655(690/60)	LP760	Park et al., 2014	
		rh-237	Synthetic	Hg/Xe arc lamp	(560/55)	LP650	Li et al., 2017	Wang et al., 2017
		rh-421	Synthetic	Halogen lamp	(565/24)	630/69	Streit and Kleinlogel, 2018	
		rh-1691	Synthetic	—	—	—	Zaglia et al., 2015	
		BeRST1	Synthetic	LED	635(630/30)	LP665	Streit and Kleinlogel, 2018	
		di-4-ANEPPS	Synthetic	Halogen lamp	(525/50)	LP600	Watanabe et al., 2017	Bingen et al., 2014
		QuasAr 1	GEVI	Laser	593.5	LP665	Streit and Kleinlogel, 2018	
		QuasAr 2	GEVI	Laser	640	660-760	Dempsey et al., 2016	Björk et al., 2017
		Arch(D95N)	GEVI	Laser	647		Björk et al., 2017	
		**Calcium Sensors**		
		rhod-2AM	Synthetic	LED	530(535/50)	570-625	Klimas et al., 2016	Jia et al., 2011
		rhod-4AM	Synthetic	LED	530	LP565	Wang et al., 2017	
		GCaMP5f	GECI	Laser	488		Björk et al., 2017	
		GCaMP6f	GECI	Laser	488		Dempsey et al., 2016	
		**Voltage Sensors**		
ArchT (Hyperpolarizing)	566	QuasAr 1	GEVI	Laser	593.5	LP665	Streit and Kleinlogel, 2018	
		**Voltage Sensors**		
eNpHR3.0 (Hyperpolarizing)	590	PGH1	Synthetic	LED	655(690/60)	LP760	Park et al., 2014	


*Opsin excitation wavelengths specified in the table are actual excitation wavelengths used in referenced studies. Sensor excitation wavelengths are shown as light source maxima with filtering shown in brackets. Bandpass filters shown as CWL/FWHM. LP, longpass.*

Synthetic sensors will non-specifically stain all the cell subpopulations in cardiac preparations. In contrast, genetic sensors can be successfully utilized *in vivo* and *in vitro* to cell specifically stain cardiomyocytes (or other cell types) (Hou et al., 2014; Dempsey et al., 2016). They hence allow long-term, cell specific, imaging of electrical and calcium activity, currently not possible with synthetic sensors. Furthermore, synthetic sensors suffer from internalization, phototoxicity and cytotoxicity making them only suitable for short-term imaging of cardiac preparations (Kaestner et al., 2015). The extent to which synthetic sensors exhibit toxicity is dependent on study specific parameters including experimental model, sensors concentration and illumination protocols. For example, di-4-ANBDQBS has been shown to exhibit little phototoxicity even at high illumination intensities (Kanaporis, 2012). In this case, the sensor was loaded into guinea pig ventricular preparations and illumination time was restricted to 1 min. In other studies, continuous illumination of di-4-ANBDQBS for 10 min showed significant phototoxic effects compared to a genetic sensor in both human induced pluripotent stem cell cardiomyocyte monolayers (Shaheen et al., 2018), and single cells (Streit and Kleinlogel, 2018). Thus, both of these studies suggest that despite their slower response kinetics, GEVIS and GECIs are valuable tools not only for *in vivo* study but also in situations where phototoxic effects are prominent, such as cellular monolayers and single cells (Fast and Kléber, 1994; Broyles et al., 2018; Streit and Kleinlogel, 2018).

## Designing an All-Optical Setup

The development of a successful all-optical system relies on a number of key features, unique when compared to single purpose, single cell, optical mapping or optogenetic setups. Light must be delivered to both the opsins and the voltage or calcium sensors, demanding distinct illumination characteristics. The possibility of illumination crossover necessitates careful consideration of sensors, actuators and filtering requirements. Table 1 summaries opsin-sensors combinations that allow stimulation of optical actuation, control and imaging in pivotal all-optical studies.

### Opsin and Sensor Compatibility

**FIGURE 3:**
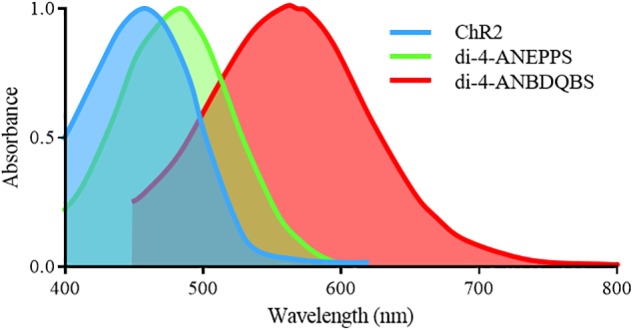
Excitation spectra of the depolarizing opsin ChR2-H134 and voltage sensitive sensors di-4-ANEPPS and di-4-ANBDQBS. Significant overlap exists between the excitation spectra of ChR2 and di-4-ANEPPS, preventing imaging of di-4-ANEPPS without perturbing the membrane potential of ChR2 expressing cells. However, the red-shifted spectra of di-4-ANBDQBS does allow for excitation without simultaneous activation of ChR2.

For an all-optical system, the compatibility of opsins with voltage and Ca^2+^ sensors is paramount, as spectral overlap will result in unwanted crosstalk and perturbation of the cellular membrane. For example, the absorption spectra of the most commonly used synthetic voltage sensor di-4-ANEPPS (excitation peak = 475nm) overlaps with that of ChR2, Figure 3. Therefore, excitation of di-4-ANEPPS to optically measure voltage can also excite ChR2, perturbing the membrane potential (Park et al., 2014). With this spectral congestion in mind, it is not surprising that the pioneering all-optical setups imaging Ca^2+^ routinely used rhod-4AM (Jia et al., 2011), which exhibit less spectral overlap with ChR2.

The use of opsins or sensors with red-shifted absorption profiles is the most common solution to spectral congestion. In general, it is easier for an all-optical system to utilize a red-shifted sensor, as the further red-shift of fluorescence emission can then be simply long-pass or band-pass filtered before imaging. Pittsburgh I (PGH1) is a potentiometric sensor whose absorption and emission spectra are far red shifted in comparison to di-4-ANEPPS, with an excitation and emission peak of 608 and 880 nm respectively (in EtOH) (Salama et al., 2005). It was hence the first sensor used in an all-optical manner with concurrent opsin excitation and voltage mapping (Park et al., 2014). By allowing opsin excitation with simultaneous voltage mapping, this system demonstrated how optogenetics can be used to not only pace cardiac tissue but crucially also prolong the action potential by ChR2 excitation during repolarization, a potential therapeutic approach (Karathanos et al., 2014), or silence activity by eNpHR3.0 driven hyperpolarization (Park et al., 2014).

Alongside PGH1, there are red-shifted variants of di-4-ANEPPS. They include di-4-ANBDQPQ and di-4-ANBDQBS, which share the same basic structure as di-4-ANEPPS but with a distinct chromophore and longer linker sizes. The result is red-shifted absorption and emission spectra, both effectively excited between 500 and 700 nm (Figure 3) and imaged from 700 to 900 nm (Matiukas et al., 2007). They are hence spectrally distinct from ChR2. Additionally, ‘blue-shifted’ opsins are being developed (Lam et al., 2017) which may help avoid spectral overlap, however, their use may be limited by tissue damage and penetration depth.

PGH1, di-4-ANBDQPQ, di-4-ANBDQBS and rh-1691 have all been successfully used in all-optical setups due to their favorable spectral properties. Table 1 lists potentiometric and calcium sensors used concurrently with optogenetic control and gives examples of illumination and filter setups (discussed in more detail later) that have been employed to minimize actuator-sensor crosstalk. Despite spectral overlap, sensors such as di-4-ANEPPS and GCaMP based calcium indicators have also been used in all-optical setups. However, dual-excitation, and fluorescence baseline shifts on pulse excitation, result in the requirement for spatial separation between excitation and emission areas, or need for extensive pre- and post-acquisition filtering (Dempsey et al., 2016; Li et al., 2017; Wang et al., 2017).

### Illumination Sources

For optical mapping, sensor excitation is most commonly achieved using LEDs which benefit from narrow wavelength spectra, long operational lifetimes and low heat emission (Beacher, 2008), though Tungsten-Halogen lamps, Mercury/Xeon arc lamps and lasers are also used. Illumination source is chosen based on a number of characteristics, including wavelength and power. However, illumination of the sample in terms of spatial and temporal homogeneity is of paramount importance for successful optical mapping. In contrast, optical actuation in optogenetics routinely requires impulse-like signals (temporal inhomogeneity) delivered to a specific area of the sample (spatial inhomogeneity). Additionality, a number of studies have demonstrated the importance of patterned light delivery to drive conduction dynamics and realize effective arrhythmia termination (Burton et al., 2015; Crocini et al., 2016; Feola et al., 2017). Therefore liquid crystal (Schmieder et al., 2017) and digital micromirror device (DMD) spatial light modulation coupled to an illumination source such as an LED, able to uniquely deliver synchronous patterned illumination, have been extensively used in cardiac optogenetics from its infancy (Arrenberg et al., 2010). DMDs utilize several hundred thousand or millions of hinged micrometer sized mirrors to deliver illumination patterns with high spatial resolution, an ability that has been key to several optogenetic based discoveries (Scardigli et al., 2018).

Furthermore, fiber-optic coupled LED (Prando et al., 2018) and laser-based approaches have been utilized. This has partially been driven by the potential to incorporate illuminating fibers in clinically used tools for precise spatial illumination *in vivo* (Klimas and Entcheva, 2014). Devices incorporating micro LED illumination with photodetectors have also been developed (Xu et al., 2014), and further optimization of such unique light delivery strategies is crucial if latent clinical benefits of optogenetics are to be realized.

### Optical Filtering

**FIGURE 4:**
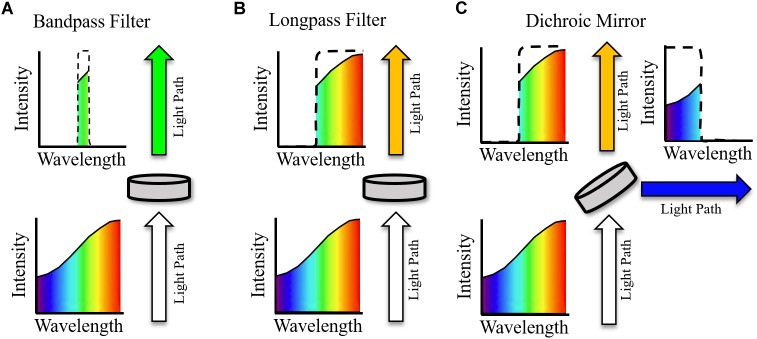
Example effect of idealized optical filters on ‘white’ light. Arrows indicate path of light with indicated spectra pre and post filtering. **(A)** Bandpass filter. All wavelengths of light, other than those within the transmission window are absorbed. **(B)** Longpass filter. Light with wavelengths above the central wavelength (CWL) are transmitted, and all other wavelengths are absorbed. **(C)** Dichroic mirror. As with the longpass filter, light with wavelengths above the CWL are transmitted. Light with wavelengths below the CWL is reflected at an angle of incidence, commonly 45^°^.

The use of compatible opsins and sensors to avoid spectral overlap has already been discussed. However, optical filtering is still crucial in effective excitation and imaging of samples, especially when considering the multiple light paths necessary in the majority of all-optical setups. Single wavelength filters utilized in all-optical setups can be broadly characterized as (i) Bandpass filters (ii) Long- or shortpass filters, and (iii) Dichroic mirrors/beam splitters (Figure 4). An idealized single wavelength bandpass filter (Figure 4A) absorbs wavelengths outside a defined window, characterized by a central wavelength (CWL) and full width half maximum (FWHM). A common use of bandpass filters in all optical setups is excitation filtering, where illumination light is filtered before reaching a sample to narrow spectral bandwidth with a relatively small FWHM filter. This helps avoid interference with other sensors or actuators (Jaimes et al., 2016). For example, an effective excitation filter for red shifted voltage sensor di-4-ANBDQPQ may be a 640/40 nm (CWL/FWHM) bandpass filter (Scardigli et al., 2018), placed between the illumination source and sample.

Longpass filters (Figure 4B) will absorb light with a wavelength below the CWL but will transmit light above this threshold (with the opposite holding for a shortpass filter with the same CWL). They therefore do not have an associated FWHM and are most used as emission filters, absorbing excitation light wavelengths while conserving the red-shifted fluorescence emission (Park et al., 2014). In conjunction with removing excitation light, longpass filtering is also necessary to effectively measure voltage dependent signals that arise from spectral shift, e.g., the electrochromic based indicators previously discussed, as the fluorescence must be imaged away from the emission maxima. Bandpass filters with red-shifted CWL compared to excitation wavelengths (and emission maxima if required) can also be used for emission filtering, i.e., between the sample and imaging device. For an effective emission bandpass filter, however, a large FWHM is often desirable to maximize the photon count at the imaging device, assuming absorbance of excitation light is maintained (Scardigli et al., 2018). Table 1 summaries excitation and emission filtering setups that have been implemented in all-optical setups encompassing both voltage and calcium indicators.

Bandpass and longpass filters interact with light by either absorbing or transmitting depending on wavelength. Dichroic mirrors (or beam splitters), however, reflect certain wavelengths of light. An idealized longpass dichroic mirror for example (Figure 4C) transmits light with wavelength above the CWL and reflects (often at an angle of incidence of 45^o^) wavelengths below this cut off. Therefore, aside from basic filtering that is also possible to achieve with longpass and bandpass filters, dichroic mirrors can be used for important functions such as directing light paths (Scardigli et al., 2018) and splitting emission in dual sensor setups to either separate cameras or sections of a single imaging chip (Dempsey et al., 2016; Wang et al., 2017). The filters outlined in Figure 4 are known as single wavelength or single band filters, with one characterizing CWL. In contrast, multiband filters are characterized by multiple transmission wavelength regions. They have hence found particular utility in dual optical mapping setups to simultaneously filter voltage and calcium emission signals (Wang et al., 2015).

### Removing Spectral Deconvolution Requirement

Problems with spectral overlap can be circumvented by utilizing optical imaging techniques that are not reliant on fluorescent sensors, i.e., do not require illumination. On initiation of the action potential, excitable cells exhibit changing light scattering properties, changing contrast (Cohen et al., 1972). However, with well-thought out setup design such as contrast enhancing off-axis illumination, the optical changes of the cell have shown to be a useful (although limited) substitute to direct sensor imaging of transmembrane voltage (Burton et al., 2015). Although parameters such as action potential morphology cannot be accurately recovered, sensor free imaging allows non-invasive tracking of excitation waves through cellular monolayers, and simple integration with optogenetic actuators (Burton et al., 2015). More recently, there has been development of bioluminescent, rather than fluorescent GEVIs such as the FRET based LOTUS-V (Inagaki et al., 2017). As bioluminescent output is stimulated chemically by treatment with a substrate such as Furimazine rather than optically, no illumination is required. This avoids the need for spectral separation between any excitation light and actuators present and facilitates long-term voltage imaging. When expressed in human induced pluripotent stem cell cardiomyocytes, LOTUS-V has been shown to deliver comparable, action potential signals to the synthetic sensor di-8-ANEPPS, although values such as action potential duration were prolonged due to the slower response kinetics (milliseconds) of the FRET response compared to the ‘fast’ synthetic sensors (Inagaki et al., 2017). The use of bioluminescent sensors in all-optical setups may therefore provide an attractive alternative to fluorescent GEVIs.

Additionally, novel non-genetic techniques for optical cardiac excitation based on graphene substrates (Savchenko et al., 2018), x-ray and ultrasound activated nanoparticles (Berry et al., 2015), or infrared induced temperature gradients (McPheeters et al., 2017) can act as a solution to spectral overlap and to realize deep tissue activation. These optical pacing strategies, however, do not share some of the unique advantages that genetically induced optical de- and hyperpolarization allows, most prominently precise control over wave dynamics or cell-specific activation.

### Imaging

As with many other areas of the life sciences, cardiac optical mapping has benefitted from continued advancement of camera technology. Photodiode arrays (PDAs) were utilized in the early optical mapping experiments that moved beyond single photomultiplier tubes (Salama et al., 1987). PDAs benefit from a large dynamic range (as the PDA is made up of large individual diodes) and enhanced sampling rates. Low fractional changes in sensor fluorescence and the sub millisecond dynamics of cardiac electrical activity make these invaluable features for successful optical mapping (Efimov and Salama, 2012).

However, due to the physical arrangement of individual diodes, the maximal spatial resolution of a PDA is more limited compared to charge-coupled device (CCD) and complementary metal oxide semiconductor (CMOS) cameras. In modern setups therefore, CCD and CMOS cameras dominate (Gloschat et al., 2018; Wen et al., 2018). Developments such as electron-multiplication in CCD cameras, and 2nd generation back illuminated ‘scientific’ CMOS cameras have helped improve dynamic ranges and noise levels of these cameras, while enhanced compatibility with standard computer interfaces such as USB-3 help alleviate previously prohibitive costs (Boukens and Efimov, 2014). Crucially, these cameras offer much higher spatial resolutions than possible with PDAs, capable of capturing > 10,000 pixel images at kHz sampling rates (Yu et al., 2017). The choice of imaging device for an all-optical setup does not differ considerably from a traditional optical mapping setup, and PDAs, CCD cameras and CMOS cameras have all been successfully utilized. Another important consideration is quantum efficiency of imaging devices in the far-red to near infrared wavelengths, if using red-shifted sensors.

### Dual Voltage–Calcium All Optical Setups

Optical mapping of preparations dual loaded with both voltage and calcium sensors offers unique insights into the interplay between the cardiac action potential and Ca^2+^ handling (Myles et al., 2012). Achieving dual voltage-calcium optical mapping requires careful consideration of sensors spectra and filtering requirements. Addition of optical actuation, with its unique spectral, spatial, and temporal illumination requirements, further complicates the setup. Therefore to date, optical setups utilizing both voltage and calcium sensors have been shown to image the two indicators separately or sequentially (Klimas et al., 2016). In all-optical setups with simultaneous voltage-calcium imaging and optical pacing, spatial separation was necessary between pacing site and imaged area (Dempsey et al., 2016).

### Computational Post-processing

In lieu of physical methods, computational methods can be used to overcome the spectral congestion that arises from optogenetic manipulation of optically mapped samples. However, it is important to note that these do not overcome undesired modulation of the membrane potential. A simple example is automatic identification and removal of optical pacing peaks by the application of several filters (Feola et al., 2017) or image frame removal (O’Shea et al., 2019). This is an effective strategy providing filtering/frame removal does not alter the fluorescence signal properties at pertinent times, and so ideally requires either temporal or spatial separation between pulsing peaks and fluorescence output.

Light pulses, however, can be more prohibitive to optical mapping analysis if pulses are applied during relevant phases of the cardiac cycle. For example, when pulses of light are delivered during the plateau and repolarization of the action potential, despite optical filtering attempts to avoid crosstalk, blue and green pulses can cause baseline shift in OAPs. As demonstrated by Park et al. (2014), to compensate for this, ‘gap compensation’ modeling procedures can be implemented. Briefly, this involves construction of a perturbation model of the optical emission (F_*bluepulse*_) like shown in Equation 1

Fbluepulse=Ffit+A.[e−(pulset−t)slowτ−e−(pulset−t)fastτ]2

where *F_fit_* is the model of a control action potential without blue light illumination, A is a constant, *t* is time, *t_pulse_* is start time of the light pulse, and τ*_slow_* and τ*_fast_* are decay constants. The repolarization phase of the perturbed OAP therefore is modeled by least square fitting a bi-exponential decay from the start of the blue light pulse allowing recovery of the OAP during optogenetically induced action potential prolongation (Park et al., 2014).

## Contactless Actuation and Electrophysiology

Both in basic research and clinically, electrode-based techniques for pacing and stimulation require direct or close contact to cardiac tissue. Although extensively exploited and effective, these actuation methods are not without limitation. The direct contact between electrodes and tissue can promote electrochemical reactions and reactive oxygen species formation. Conversely in some situations, for example in cardioversion strategies (Nyns et al., 2017) or in cardiomyocyte monolayers to improve signal quality (Lapp et al., 2017), simultaneous activation of the large areas is desired rather than at a single site. If the activation region needs to be changed then electrodes must be physically moved, limiting spatial flexibility and throughput. From a clinical perspective, implantable pacemaker and defibrillation devices are highly energy consuming, tissue damaging, prone to post-implantation complications and can cause psychological distress and reduced quality of life (Bruegmann et al., 2016; Israelsson et al., 2018).

Optogenetic pacing therefore is one of a few biological strategies being explored as a replacement for traditional pacemaker devices (Rosen et al., 2011) but undoubtably its most immediate beneficial quality is the ability realize unique basic research possibilities. Optogenetic opsins can be delivered to specific cell types (Hulsmans et al., 2017; Wang et al., 2017), providing novel research strategies, and promoting precise, repeatable and coordinated activation patterns. Conversely, by changing illumination conditions or the spatial arrangement of opsins expression, simultaneous excitation/suppression of large areas can be achieved. This can be applied in cellular monolayers, where concurrent activation can enhance signal quality, or in cardioversion strategies to terminate arrhythmias (Bruegmann et al., 2018). Contactless actuation avoids tissue damage due to the lack of tissue-electrode interface and can be realized in high-throughput applications such as multi-well cellular assays, discussed in more detail later (Klimas et al., 2016).

Optical mapping offers several clear advantages compared to electrode recording techniques. The spatial resolution achievable with optical mapping systems greatly outperforms multi-electrode array mapping systems. Furthermore, optical imaging can allow detailed, direct and multi-parameter investigation of voltage and calcium dynamics, whereas electrode techniques often make indirect measurements such as field potentials, where signals can be corrupted by noise and can alter significantly over time due to changes in electrode positioning or the maintenance of contact. Therefore, despite some notable disadvantages of optical study including requirement of contraction uncouplers and inability to perform *in vivo* experiments (Boukens and Efimov, 2014), optical mapping techniques have seen growing use, even prior to transformative capabilities of optical stimulation was made available. The recent combination of actuation and measurement in all-optical setups has therefore, in a relatively short timeframe, delivered remarkable insights into optogenetic pacing (Nussinovitch and Gepstein, 2015a), cell targeting (Wang et al., 2017) and arrhythmia termination (Feola et al., 2017); advancing basic understand and enhancing the prospect of future clinically relevant optical therapies.

## Effects of Light Pacing on Action Potential Morphology and Propagation

For further implementation of optogenetic technologies, detailed understanding of whether optogenetic actuation affects cardiac electrical signal morphology is required. All-optical systems are uniquely advantageous for such investigations, as they are able to report key EP signal parameters for comparison against established techniques, while also providing high spatio-temporal recording of cardiac activation (Entcheva and Bub, 2016).

Recent work using human stem cell-derived cardiomyocytes expressing commercial optogenetic constructs OptoPatch and CaViar have demonstrated that optogenetic modulation does not significantly alter single cell electrophysiological properties (Björk et al., 2017). Furthermore, optogenetic manipulation of neonatal rat ventricular myocyte monolayers expressing ChR2 indicate unaltered conduction velocity, action potential duration and upstroke velocity compared to control cells, using either optogenetic or electrical actuation (Li et al., 2017). Computational insights have for the most part agreed with experimental findings by showing similar action potential morphologies and cell type dependent variability (Williams and Entcheva, 2015). Nevertheless, fundamental differences in electrical and optogenetic stimulation exist. Electrical current injection is traditionally achieved with a rectangular pulse delivered at short pulse widths of 2–10 ms (Myles et al., 2012; Holmes et al., 2016). ChR2 photocurrent exhibits slower onset kinetics, meaning longer pulse timescales can be required to reach excitation threshold (Williams and Entcheva, 2015). The effects of these stimulation differences on *in vitro* and *in vivo* cardiac electrophysiology require further examination.

## Applications of All Optical Cardiac Electrophysiology

All-optical systems, in their relatively short period of existence, have expanded our understanding of cardiac pathophysiology thanks to their high spatio-temporal resolution and unique ability for targeted tissue excitation. Indeed, by optically mapping voltage in transgenic mice expressing ChR2, threshold excitation and vulnerable areas for proarrhythmic focal ectopic activity has been determined (Zaglia et al., 2015). All-optical setups have allowed demonstration and potential for utility of ChR2 mediated resynchronization (Nussinovitch and Gepstein, 2015a) and have crucially informed the production of local rather than global areas of conduction block using patterned illumination. The patterned illumination shows similar success in arrhythmia termination but at lower energy costs than global activation (Crocini et al., 2016; Feola et al., 2017). However, whether optogenetic approaches confer energy reduction benefits, over established and effective electrode technologies requires detailed assessment in future studies.

Previously, optical mapping of arrhythmia dynamics in whole hearts has allowed mechanistically driven choice of patterned illumination in an ‘open-loop’ fashion - information based on previous recordings being used to dictate illumination patterns. Although this proved similarly effective as global illumination, there is reliance on consentient arrhythmia dynamics between hearts (Crocini et al., 2016). However, recent advancements including utilization of DMD illumination technology and high-speed data recording have demonstrated the potential of ‘closed-loop’ all-optical EP. Here, conduction disorders such as atrioventricular block can be detected by optical mapping and corrected by optical stimulation of ChR2 in real time. Real-time intervention can also conversely be used to setup and then study conduction abnormalities such as re-entry. These abilities make ‘closed-loop’ systems potentially vital going forward, allowing all-optical research in experimental models that is tunable in a manner hitherto only afforded by computational modeling, and at much reduced timescales (Scardigli et al., 2018). Similarly, all-optical control and output has shown the feasibility of engineering bioelectric tissues capable of complex information processing and in which all constituent parts are fully characterized (McNamara et al., 2016).

A major avenue for use of all-optical setups is the delivery of high-throughput platforms for cardiotoxicity screenings of candidate drug compounds (Klimas et al., 2016; Streit and Kleinlogel, 2018). These platforms are crucial in the context of the comprehensive *in vitro* pro-arrhythmia assay (CiPA) initiative – the recognition that cardiotoxicity screening should not focus solely on hERG channel interactions and must use experimental as well as computational methods (Fermini et al., 2016). All-optical methods are distinctly suited to the changing requirements of cardiotoxicity drug screening. As highlighted, the lack of requirement for direct contact makes optical actuation and optical recording much easier to scale to high-throughput parallelized applications, crucial for screening multiple drugs. As the reported voltage signals result from the sum of all ionic currents, significant alterations in any channel or pump function (not just hERG/I_Kr_) in response to a drug will be evident, while Ca^2+^ handling abnormalities can also be screened with the use of appropriate sensors (Dempsey et al., 2016; Klimas et al., 2016). If specific channels warrant further investigation, all-optical methods can still be utilized by expression of channels in cells otherwise void of the ionic channels of interest (Streit and Kleinlogel, 2018), and the scalability of optical methods means analysis of other models apart from cell cultures may be achievable in the near future.

## Conclusion and Future Directions

Optogenetics is a technique that has developed into an immensely useful tool in basic cardiac research with clear, though as of yet unrealized, clinical potential (Entcheva, 2013). Fusion of optogenetics with optical mapping has been made possible by the substantial technical considerations and advances summarized in this review. All-optical electrophysiology, and indeed the field of cardiac optogenetics in general, however, remains a relatively new technique with several opportunities to further advance our understanding of electrical function in the heart.

Recent demonstration of ‘closed-loop’ all-optical investigation opens up a plethora of exciting opportunities for application of this exciting technology to key research questions, relevant in health and disease. ‘Closed-loop’ all-optical investigation studies could deliver faster, more physiologically relevant tools than even the most sophisticated computational cardiac models.

In contrast to cardiac research, optogenetics is widespread in neuroscience (Adamantidis, 2015). Effects on heart function and coupling of neurons and cardiac cells are often measured outcomes of neuromodulation. However, cardiac effects of optical neuromodulation are potentially limited to simply beating rate measurements (Nussinovitch and Gepstein, 2015b) or monitored using traditional techniques, with only isolated examples of optical mapping neuronal-cardiac effects (Dong et al., 2016). The combination of optical neuromodulation with all-optical cardiac electrophysiology could deliver unique insights into control of the heart by the nervous system (Wengrowski et al., 2015).

For optogenetic-based therapy to ever be realized in clinical practice, several technological and biological advances need to be implemented. These include novel methods for light and gene delivery to *in vivo* cardiac tissue, wireless control of implantable devices (Gagnon et al., 2017), and advanced materials uniquely designed for use in bio-integrated electronic circuits (Fang et al., 2016). However, regardless of future clinical utility, implementation of optogenetics in all-optical imaging systems has already proved a unique and transformative tool for cardiac research and will continue to be used in the study of the physiology and pathophysiology of the heart.

## Author Contributions

CO prepared the primary manuscript. AH, JW, JC, PK, LF, KR, and DP critically revised the manuscript. XO, RD, and SH provided intellectual content and technical insights. CO, JW, and DP produced the figures.

## Conflict of Interest Statement

The authors declare that the research was conducted in the absence of any commercial or financial relationships that could be construed as a potential conflict of interest.
